# The Association between the Use of Proton Pump Inhibitors and the Risk of Hypomagnesemia in a National Cohort of Veteran Patients with HIV

**DOI:** 10.1177/2325958218821652

**Published:** 2019-01-22

**Authors:** S. Scott Sutton, Joseph Magagnoli, Tammy Cummings, James W. Hardin

**Affiliations:** 1 Department of Clinical Pharmacy and Outcomes Sciences, University of South Carolina, College of Pharmacy, Columbia, SC, USA; 2 Dorn Research Institute, WJB Dorn Veterans Affairs Medical Center, Columbia, SC, USA; 3 WJB Dorn Veterans Affairs Medical Center, Columbia, SC, USA; 4 Biostatistics Division, Department of Epidemiology & Biostatistics, University of South Carolina, Columbia, SC, USA

**Keywords:** HIV, hypomagnesemia, proton pump inhibitors

## Abstract

**Objectives:**

To examine the risk of hypomagnesemia of HIV-positive patients adherent to proton pump inhibitors (PPIs).

**Methods:**

A cohort study utilizing the Veterans Affairs Informatics and Computing Infrastructure was conducted on patients with (1) a complete antiretroviral therapy, (2) a serum magnesium measure during the study period, and (3) adherent to PPIs. Statistical analyses evaluated baseline characteristics between cohorts and a Cox proportional hazards model evaluating the association of hypomagnesemia while adjusting for baseline covariates.

**Results:**

A total of 6047 patients met the study inclusion criteria, 329 patients in the PPI cohort and 5718 patients in the non-PPI cohort. The stratified Cox proportional hazards model results revealed that the risk of hypomagnesemia for the PPI cohort is 3.16 times higher compared to the non-PPI cohort (adjusted hazard ratio = 3.16, 95% confidence interval = 2.56-3.9).

**Conclusions:**

Proton pump inhibitors medication usage in HIV-positive patients is associated with a higher risk of hypomagnesemia compared to non-PPI patients.

**What Do We Already Know about This Topic?**

Preclinical and clinical studies have shown that proton pump inhibitors (PPI) usage is associated with hypomagnesemia; however, select studies refute the association of hypomagnesemia to PPIs.

**How Does Your Research Contribute to the Field?**

Research in select patient groups can help answer the association or lack of between PPIs and hypomagnesemia; specifically, what is the relationship between PPI usage and hypomagnesemia within an HIV population receiving antiretrovirals?

**What Are Your Research’s Implications toward Theory, Practice, or Policy?**

The research implications are for practice; specifically, this research can add clinicians in prescribing of proton pump inhibitors in patients with HIV and assist with monitoring if PPIs are utilized.

## Introduction

Proton pump inhibitors (PPIs) are extensively utilized in the United States and available as prescription and over the counter (OTC) to treat gastrointestinal disorders.^1,2^ Proton pump inhibitors usage is generally considered safe; however, mounting evidence has been generated that raises concern about adverse effects, including hypomagnesemia.^1,2^ Proton pump inhibitors interfere with the absorption path of active intestinal Mg^2+^, and the Food and Drug Administration (FDA) issued a warning in March 2011 that suggests PPIs may lower serum magnesium levels for patients utilizing it for a long term.^2^ Normal magnesium levels are between 1.7 and 2.2 mg/dL, and hypomagnesemia occurs when the level of serum magnesium absorbed is below the threshold of 1.7 mg/dL.

Preclinical and clinical studies have shown that PPI usage is associated with hypomagnesemia^3–5^; however, select studies refute the association of hypomagnesemia with PPIs.^6,7^ The discrepancy in published studies warrants further research in this area to inform clinicians. Additionally, research in select patient groups can help answer the association or lack of association between PPIs and hypomagnesemia. Specifically, what is the relationship between PPI usage and hypomagnesemia within an HIV population receiving antiretrovirals? This question is of critical importance to practicing clinicians because hypomagnesemia in hospitalized HIV-positive patients is a risk factor for nonrecovery of renal function and for in-hospital mortality.^8^ To answer this question, we conducted a cohort study within the largest provider of HIV care in the United States, the Veterans Health Administration (VHA). The objective of the study is to evaluate the risk of hypomagnesemia in HIV-positive patients adherent to PPI medications compared to HIV-positive patients never prescribed PPIs among veterans in the United States Department of Veterans Affairs.

## Methods

### Data Source

This retrospective cohort study evaluating the risk of hypomagnesemia among HIV-positive patients using PPI was conducted using data from the Department of Veterans Affairs during the study period January 1, 2005, to December 31, 2013. The Veterans Affairs Informatics and Computing Infrastructure was utilized to obtain individual-level information on demographics, administrative claims, and pharmacy dispensation. The completeness, utility, accuracy, validity, and access methods are described on the Veterans Affairs (VA) website, http://www.virec.research.va.gov.

### Study Design

For inclusion into the study, patients were required to meet 3 inclusion criteria: (1) patients must have an HIV diagnosis during the study period, (2) patients must have a complete antiretroviral therapy (ART) regimen during the study period, and (3) patients must have a serum magnesium measure while on ART. A complete ART regimen was defined as 2 nucleoside/nucleotide reverse transcriptase inhibitors plus a third agent (ie, non-nucleoside reverse transcriptase inhibitor, protease inhibitor, integrase inhibitor). Patients included in the initial sample were then grouped into 2 mutually exclusive cohorts, HIV-positive patients prescribed PPI medications and HIV-positive patients never prescribed PPIs during the study period. The study medications included omeprazole, pantoprazole, lansoprazole, rabeprazole, esomeprazole, dexlansoprazole, and omeprazole/sodium bicarbonate. The study index date was defined as the first date, within the study period, in which the patient is on a complete ART, has a serum magnesium measure, and is on one of the study PPI medications (for the PPI cohort). The index magnesium level was measured while patients were on a PPI.

To examine the effect of prescribed PPIs while on an ART regimen, we only included patients in the cohort who had no previous PPI prescriptions within 6 months prior to index. Further, to capture true effect of the study medication, only HIV-positive patients with a PPI adherence rate greater than or equal to 80% were included in the study. Proton pump inhibitor adherence was measured by the proportion of days covered (PDC). The PDC was calculated as the number of days the patient had the PPI available divided by the total time in the study. The 80% adherence threshold is consistent with other chronic diseases and is categorized as adherence with chronic diseases.^9^ Patients were followed from the index date until the earliest date of:

1. Hypomagnesemia defined as magnesium level less than 1.7 mg/dL
2. End of study period, December 31, 2013
3. Death

All magnesium levels were evaluated within the study period and flagged if low. Therefore, only onetime point was evaluated without follow-up for patients with low magnesium levels. Patients who did not develop hypomagnesemia after index were considered censored at follow-up.

### Study Variables

This study examines the risk of hypomagnesemia within a national cohort of HIV-infected Veterans receiving ART. The hypomagnesemia study outcome was created by pulling all serum magnesium levels from the VA laboratory data using a Logical Observation Identifiers Names and Codes (LOINC) code of 19123. Hypomagnesemia was flagged as serum magnesium levels <1.7 mg/dL. Several covariates were utilized to account for differences among the patients (demographic, disease burden, and laboratory). Demographic variables included age, sex, and race coded as white, black, and other/unknown. The Charlson comorbidity index, excluding AIDS diagnoses, was utilized to account for differences in disease burden.^10^ Additionally, select baseline variables were accounted for and included serum magnesium level and viral load suppression at index. A measure of viral load suppression based on the results of the patient’s viral load count at the closest point to index was created. Because HIV RNA levels were determined using assays with varying detection limits, values < 400 copies/mL were used to define viral suppression.^11,12^ Values > 400 copies/mL are considered not virally suppressed. Patient’s without viral load counts within 1-month pre- or post- index were considered to have unknown viral suppression.

### Statistical Analysis

The statistical analysis occurred in 2 steps. The initial step utilized statistical tests to compare baseline characteristics between the PPI and non-PPI cohorts. For continuous variables such as age, serum magnesium, and the Charlson comorbidity index, a Wilcoxon rank sum test was utilized. Categorical variables were analyzed using the χ^2^ test. In the second step, a stratified Cox proportional hazards model was utilized to evaluate the association between PPIs and hypomagnesemia while adjusting for baseline covariates. Proportionality of the hazards was checked using the scaled Schoenfeld residuals.^13^ After stratifying the model by baseline hypomagnesemia (index Mg <1.7 mg/dL yes/no), no violations of proportionality were found.

### Research Ethics and Patient Consent

The study was conducted in compliance with the Department of Veterans Affairs requirements, received institutional review board (IRB) and Research and Development approval. The IRB approval was expedited because of the use of existing data via a claims data base study; therefore, a Health Insurance Portability and Accountability Act (HIPAA) waiver was granted.

## Results

A total of 6047 patients were identified in the VHA data set that met all the study criteria and comprised the initial sample. Table 1 displays the sample characteristics at index for the PPI and non-PPI cohorts. There were 5718 HIV-positive patients without a PPI prescription and 329 HIV-positive patients prescribed a PPI. On average, the PPI cohort was (1) older than the non-PPI cohort (55 and 51 years old, respectively, *P* < .001), (2) 39% black compared to 43% black (*P* < .001), and (3) 90% male compared to 87% (*P* = .021). The PPI cohort had a lower percentage of patients virally suppressed at baseline (29% compared to 37%, *P* = .017), a higher Charlson comorbidity index (average Charlson comorbidity of 2.66 compared to 1.01, *P* < .001), and a higher percentage of patients with a history of drug/alcohol use (47% compared to 38%, *P* = .003). Index magnesium levels were statistically significantly different (*P* < .001). Additionally, the PPI cohort had 9% patients with an index magnesium level less than 1.7 mg/dL at baseline compared to 6% of the non-PPI cohort (*P* = .007). Antiretroviral therapy utilization within the study consisted of single tablet regimens and multitablet regimens (Table 1). The PPI cohort has less patients receiving a protease inhibitor (46% versus 52%, *P* = .038) and more patients receiving a multitablet non-nuceloside reverse transcriptase inhibitor (26% versus 36%, *P* < .001).

**Table 1.** Sample Characteristics at Index.

Variables	Non-PPI Control, N = 5718	PPI, N = 329	*P* Value
Age at index (years)	51.08 (10.151)	55.01 (9.428)	<.001
Race			
Black	2484 (43%)	127 (39%)	<.001
Other/Unknown	1480 (26%)	68 (21%)	
White	1754 (31%)	134 (41%)	
Sex			
Female	127 (2%)	11 (3%)	.021
Male	4976 (87%)	297 (90%)	
Unknown	615 (11%)	21 (6%)	.021
Viral suppression			
No	1056 (18%)	67 (20%)	.017
Unknown	2550 (45%)	166 (50%)	
Yes	2112 (37%)	96 (29%)	
Charlson comorbidity	1.01 (1.564)	2.66 (2.851)	<.001
Drug/alcohol	2200 (38%)	154 (47%)	.003
Index Mg (mg/dL)	2.06 (0.279)	1.99 (0.278)	<.001
Index Mg <1.7 mg/dL	325 (6%)	31 (9%)	.007
Year	2007.3 (3.554)	2007.39 (3.068)	.649
Regimen^a^			
STR	1299 (23%)	65 (20%)	.237
MTR: PI	2952 (52%)	150 (46%)	.038
MTR: NNRTI	1497 (26%)	120 (36%)	<.001
MTR: ISTI	277 (5%)	24 (7%)	.063

Abbreviations: ISTI, integrase strand transfer inhibitors; MTR, multiple-tablet regimen; NNRTI, non-nucleoside reverse transcriptase inhibitor; PI, protease inhibitor; PPI, proton pump inhibitors; STR, single-tablet regimen.

^a^Regimen percentages do not add up to 100% due to regimen switches during the study.

The most frequently prescribed PPI (Table 2) was omeprazole (82%) followed by rabeprazole (10%), lansoprazole (5%), and pantoprazole (2%). Table 3 displays the results of the stratified Cox model. The results reveal that the risk of hypomagnesemia for the PPI cohort is 3.16 times higher compared to the non-PPI cohort (hazard ratio [HR] = 3.16, 95% confidence interval [CI] = 2.56-3.9). Several covariates were accounted for in the model. There was no statistically difference for the covariates of age and sex. However, select covariates were statistically significant within the model and included race, Charlson comorbidity index, viral suppression, drug and alcohol history, and index year. Specifically, the covariates in the Cox model demonstrate that (1) white patients have a lower risk of hypomagnesemia compared to black patients (HR = 0.6528, 95% CI = 0.56-0.76), (2) a 1-unit increase in the Charlson comorbidity index increases the risk of hypomagnesemia by 19% (HR = 1.1932, 95% CI = 1.16-1.23), (3) virally suppressed had a lower risk of hypomagnesemia (HR = 0.5546, 95% CI = 0.46-0.67), and (4) patients with a drug and or alcohol diagnosis in the year prior to index had a higher risk of hypomagnesemia (HR = 1.4215, 95% CI = 1.24-1.63).

**Table 2.** Proton Pump Inhibitors Drug Frequencies.

Drug	N	%
Omeprazole	270	82
Rabeprazole	34	10
Lansoprazole	18	5
Pantoprazole	7	2

**Table 3.** Risk of Hypomagnesemia (Magnesium < 1.7 mg/dL).

Variables	HR^a^	95% CI
PPI 80%-100% PDC	3.1624	2.56-3.90
Age (18-25 years reference)		
26-35	0.9731	0.23-4.07
36-45	1.3318	0.33-5.39
46-55	1.4434	0.36-5.83
56-65	1.9677	0.49-7.98
66-75	1.694	0.41-7.04
75+	3.779	0.88-16.31
Sex (female reference)		
Male	0.7865	0.50-1.23
Unknown	0.7951	0.48-1.30
Race (black reference)		
Other/unknown	0.7977	0.66-0.97
White	0.6528	0.56-0.76
Charlson comorbidity	1.1932	1.16-1.23
Virally suppressed (no reference)		
Unknown	0.7691	0.65-0.90
Yes	0.5546	0.46-0.67
Drug/alcohol	1.4215	1.24-1.63
Year	0.9405	0.92-0.96

Abbreviations: HR, hazard ratio; PDC, proportion of days covered; PPI, proton pump inhibitors.

^a^Model is stratified on indicator for index magnesium (<1.7 mg/dL yes/no); N: 6047; No. Events: 901; H_o_: Proportional Hazards holds: χ^2^ = 23.02, *P* value = .11.

## Discussion

Magnesium is the fourth most abundant intracellular ion and has numerous essential functions in intracellular metabolism and ion transport. Total body magnesium is primarily housed within bone cells, while the remaining 1% circulates in the blood. As with most electrolytes, the balance of intake, absorption, excretion in the gastrointestinal and renal systems, and the constant flux between the circulating and storage compartments within the serum and bone are the determinants of magnesium homeostasis. The association between PPI utilization and hypomagnesemia was first recognized through a case report published in 2006.^14^ Initial reports describe patients with chronic PPI exposure, presenting with symptoms characteristic of hypomagnesemia, including arrhythmias and symptoms of neuroexcitability such as seizures and tetany.^14,15^ Since then, several preclinical and clinical studies have confirmed the association of PPI exposure and serum magnesium concentrations.^1,4,5,14–20^ Studies demonstrate a classwide PPI effect of hypomagnesemia and discontinuation results in recovery and rechallenge has led to reoccurrence.^21^ However, not all studies have validated the PPI risk of hypomagnesemia finding.^6,7^ We conducted a PPI study to add to the hypomagnesemia literature and to evaluate a specific patient population (HIV). The Department of Veterans Affairs is the largest provider of HIV care within the United States, and PPI use is very common among Veterans. Proton pump inhibitors have also demonstrated an increased overall mortality risk in the VA.^22^ Additionally, gastric acid-reducing agents have been reported as frequently prescribed in HIV-positive patients receiving antiretrovirals. Therefore, the VA data are relevant to answer the association of PPIs and hypomagnesemia, and HIV-positive patients are an excellent group of patients. This retrospective analysis of United States Veterans compared HIV-positive patients prescribed and adherent to PPIs to HIV-positive patients never prescribed PPIs. The goal of this study was to assess the impact of PPI usage on the risk of hypomagnesemia. Medication adherence (or lack of) can significantly impact the association and findings; therefore, this study only evaluated patients prescribed and adherent to the PPI. If a patient were prescribed a PPI but not adherent, a claims study may not be able to identify the association. This study found that the risk of hypomagnesemia for the PPI cohort was 3 times higher compared to the non-PPI cohort.

The outcomes in our study are consistent with other studies evaluating a non-HIV cohort. The use of PPI was found to be associated with hypomagnesemia in hospitalized adult patients and within a cross-sectional study of reported adverse reactions from the FDA database showing higher risk in males and older populations.^20,23^ A Canadian population-based case–control study found that current PPI usage was associated with a 43% increase in risk of hypomagnesemia over a 10-year period among patients also receiving diuretics.^19^ Similarly, in a retrospective study of 112 patients who used PPIs, there was a statistically significant difference in lower serum magnesium levels compared to the nonmatched control group.^18^ Misra et al conducted a single-center cross-sectional design study using observational data on hemodialysis patients in Canada and concluded that PPI users had significantly lower serum magnesium levels compared to non-PPI users using unadjusted and adjusted analyses.^16^ Additionally, in a large hospital-based cross-sectional study, PPI use combined with a diuretic use was associated with hypomagnesemia within a sample of patients admitted to the intensive care unit.^17^

A 2015 meta-analysis of observational studies also suggests that PPI use is associated with hypomagnesemia and may impact clinical management.^3^ The meta-analysis evaluated 9 observational studies with discordant results (5 studies showed an association and 4 studies did not show an association). Additionally, a 2014 meta-analysis demonstrated that PPI use increases the risk of hypomagnesemia (pooled odds ratio (OR) = 1.775, 95% CI = 1.077-2.924); however, significant heterogeneity among the included studies was present implying that the effect of PPI use on hypomagnesemia varied. Among the published PPI and magnesium data, the studies consisted of vastly different populations (eg, inpatient, outpatient, intensive care, hemodialysis). Additionally, there has been a lack of adherence monitoring within the studies; therefore, the role of adherence could significantly impact the found heterogeneity.

In addition to the clinical studies discussed above, preclinical studies have identified an association between PPIs and hypomagnesemia. A rat animal model demonstrated that 24 weeks (24 weeks of rat time equates to 10 human years) of omeprazole treatment significantly suppressed plasma magnesium levels, urinary magnesium excretion, and bone and muscle magnesium content.^24^ However, 4-week omeprazole administration in mice and rats had no effect on the plasma magnesium level. Another preclinical, mice supplemented with omeprazole had significantly reduced serum magnesium levels.^25^

There are studies that demonstrate that our finding of hypomagnesemia is not generalizable to the entire population. In hemodialysis patients, PPI use did not affect serum magnesium levels. This study is specifically contradictory to the study by Misra et al.^7,16^ A pre/post- study evaluating 209 study participants found no association between PPI use and risk of hypomagnesemia among patients in the Republic of Kosovo.^6^

Given the body of evidence surrounding PPIs, the risk of hypomagnesemia is evident. Although, there could be select patient types that are not at risk. This association is important for clinicians as they develop management and monitoring plans for their patients. Specifically, clinicians should consider hypomagnesemia as a differential diagnosis for patients taking PPIs with unexplained muscle symptoms (eg, weakness, tremors, twitches), irritability, insomnia, numbness/tingling, severe confusion, irregular heart rate, or seizure. Additionally, this finding is very important for HIV-positive patients. The use of PPIs is high in the general patient population; however, HIV-positive patients have additional potential indications for PPI use. HIV-positive patients may experience diarrhea due to the ART, and the diarrhea may cause or exacerbate magnesium loss.^8,26–28^ One study states over half HIV-positive patients experience diarrhea, and a Brazilian study showed that 29% of patients on ART were at a high risk of developing hypomagnesemia.^8,26^ Given our sample size, we believe the data are robust in evaluating the risk of hypomagnesemia in PPI patients versus non-PPI patients and can assist clinicians in monitoring patients on PPIs. Our study is unique because it (1) evaluated a national cohort of patients, (2) studied a specific disease state (HIV), and (3) only evaluated patients who were adherent to the PPI. Studies that have not confirmed the association between hypomagnesemia and PPIs could simply be because patients were not adherent to medications. However, our study exhibits limitations common to observational claims database analyses. Adherence was measured from filled prescriptions; however, studies have suggested that pharmacy refill rates are a good depiction for actual medication adherence.^29^ Our study population was predominantly white, middle-aged males with HIV; therefore, our findings may not be generalizable to patients of different age groups or races. Additionally, because patients were not randomized to the different treatments, we cannot exclude unmeasured confounding factors that may have influenced our outcomes (eg, diarrhea). The lack of randomization and unmeasured confounding could account for the differences within our study. Although we attempted to control for select variables through use of multivariable models, residual confounding may remain. Specifically, medications were not evaluated in the statistical models, and medication utilization could have an impact on magnesium levels. Of the medication evaluated (PPIs), the length of duration of the PPI was not evaluated. Additionally, the covariate viral suppression was created by dichotomizing the results of patient’s last viral load count found during the ART treatment period as less than 400 copies/mL. Because of our research question and study methods, a patient may have had a viral blip during the study period. Therefore, it is possible that patients with a measurable increase in viral load were a viral blip and the patient later returned to viral suppression. Further, our study period evaluated ARTs from 2006 to 2013. Since 2013, there have been new ARTs developed and new fixed dose combinations available. Additionally, Department of Health and Human Services (DHHS) treatment guideline recommendations have changed several times since 2006. Finally, patients may have taken PPI as an OTC medication and our claims database is not able to capture OTC medications.

## Conclusion

A nationwide cohort of United States Veterans demonstrated that HIV-positive patients adherent to PPIs have an increased risk of hypomagnesemia. Future research should be considered in understanding the risks and benefits of PPI usage and the occurrence of hypomagnesemia.
